# Unexpected Regulatory Role of CCR9 in Regulatory T Cell Development

**DOI:** 10.1371/journal.pone.0134100

**Published:** 2015-07-31

**Authors:** Heather L. Evans-Marin, Anthony T. Cao, Suxia Yao, Feidi Chen, Chong He, Han Liu, Wei Wu, Maria G. Gonzalez, Sara M. Dann, Yingzi Cong

**Affiliations:** 1 Department of Microbiology and Immunology, University of Texas Medical Branch, Galveston, Texas, United States of America; 2 Department of Pathology, University of Texas Medical Branch, Galveston, Texas, United States of America; 3 Department of Gastroenterology, The Shanghai Tenth People’s Hospital, Tongji University, Shanghai, China; 4 Department of Gastroenterology, The Qilu Hospital, Shandong University, Shandong, China; 5 School of Medicine, University of Texas Medical Branch, Galveston, Texas, United States of America; 6 Department of Medicine, University of Texas Medical Branch, Galveston, Texas, United States of America; Rush University Medical Center, UNITED STATES

## Abstract

T cells reactive to microbiota regulate the pathogenesis of inflammatory bowel disease (IBD). As T cell trafficking to intestines is regulated through interactions between highly specific chemokine-chemokine receptors, efforts have been made to develop intestine-specific immunosuppression based on blocking these key processes. CCR9, a gut-trophic chemokine receptor expressed by lymphocytes and dendritic cells, has been implicated in the regulation of IBD through mediating recruitment of T cells to inflamed sites. However, the role of CCR9 in inducing and sustaining inflammation in the context of IBD is poorly understood. In this study, we demonstrate that CCR9 deficiency in effector T cells and Tregs does not affect the development of colitis in a microbiota antigen-specific, T cell-mediated model. However, Treg cells express higher levels of CCR9 compared to those in effector T cells. Interestingly, CCR9 inhibits Treg cell development, in that CCR9^-/-^ mice demonstrate a high level of Foxp3^+^ Tregs, and ligation of CCR9 by its ligand CCL25 inhibits Treg cell differentiation *in vitro*. Collectively, our data indicate that in addition to acting as a gut-homing molecule, CCR9 signaling shapes immune responses by inhibiting Treg cell development.

## Introduction

Inflammatory bowel disease (IBD) is comprised of ulcerative colitis and Crohn’s disease, which differ in the anatomical distribution of lesions, depth of tissue inflammation, and the CD4^+^ T cell subsets involved [1,2]. Accumulating evidence indicates that multiple factors regulate the pathogenesis of IBD, including genetic predisposition, environmental factors, and inappropriate immune responses to microbiota [2–4]. Among effector T cells (Teffs), both T helper (Th) 1 and Th17 cells, which produce IFNγ and IL-17, respectively, have been implicated in the pathogenesis of experimental colitis, whereas regulatory T cells (Tregs) inhibit colitis development [5–7]. Tregs express the transcription factor forkhead box P3 (FoxP3) and control inflammation through the production of TGF-β and IL-10 [8–10]. As T cell trafficking to the intestines is regulated through interactions between highly specific chemokine-chemokine receptor pairs, efforts have been made to develop intestine-specific immunosuppressive medications based on blocking these key processes [11,12].

Chemokine (C-C motif) receptor 9 (CCR9) is a gut-trophic chemokine receptor expressed by lymphocytes and dendritic cells [13–15]. CCR9 binds non-promiscuously to its ligand Chemokine (C-C motif) ligand 25 (CCL25), which is expressed in the small intestine and thymus [14–17]. During intestinal inflammation, both intestinal expression of CCL25 and recruitment of CCR9^+^ T cells are increased in cases of experimental colitis and in patients with Crohn’s disease [12,18,19]. This finding may indicate that CCL25-CCR9 interaction mediates the recruitment of T cells to inflamed sites, which could contribute to the progression of colitis. However, the role of CCR9 in inducing and sustaining inflammation in the context of IBD is poorly understood. Conflicting data implicate CCR9 as being critical to both development of pathological inflammation and protection by Tregs [12,15–17,20–22]. Furthermore, the efficacy of CCR9 blockade is dependent on the disease phase, with anti-CCR9 antibodies attenuating early, but not late, disease [12,21].

There have been few studies regarding possible non-chemoattractant functions of CCR9, mainly in cancer models. These studies indicate that ligation of CCR9 to CCL25 induces anti-apoptotic signaling and prevents chemotherapy-induced cell death in cancer cells [23,24]. While it has long been known that some chemokine receptors are preferentially expressed on specific CD4^+^ subsets, studies indicate that binding of chemokine receptors to specific ligands can directly influence T cell polarization and phenotype [25–29]. Chemokine (C-X-C motif) ligand 12 (CXCL12) ligation drives an anti-inflammatory response in both macrophages and CD4^+^ T cells, resulting in the upregulated production of IL-10, but not of FoxP3, and decreased production of IFN-γ and TNF-α [25,27,28]. Likewise, CXCL11 ligation induces regulatory Tr1 and Th2 CD4^+^ T cell phenotypes via mTOR, and treatment with a stable CXCL11-Immunoglobulin construct results in IL-10-dependent rescue from experimental autoimmune encephalitis (EAE) [26]. In contrast, CXCL10 ligation induces an inflammatory response characterized by increased CD4^+^ T cell expression of IFN-γ, IL-17, T-bet, and RORγt [26].

In studying the role of CCR9 in microbiota-reactive, T cell-mediated colitis, we unexpectedly demonstrated that CCR9 can inhibit Treg cell development. In our study, CCR9^-/-^ mice demonstrated increased Foxp3^+^ Treg populations, and ligation of CCR9 by CCL25 inhibited Treg cells *in vitro*. Collectively, our data indicate that, in addition to acting as a gut-homing molecule, CCR9 inhibits the Treg polarization, thereby contributing to its role in colitis development.

## Materials and Methods

### Mice

CBir1 flagellin-specific TCR transgenic mice (CBir1 Tg) were maintained in the animal facilities of the University of Texas Medical Branch. CCR9^-/-^ mice were kindly provided by Dr. Joshua Farber of NIAID, NIH, and crossed with CBir1 Tg mice. Tail snips were collected 3 weeks of age to confirm genotyping. Littermates were selected and cohoused until they were 6 to 8 weeks old, when experiments were performed. TCRβxδ^-/-^ mice were purchased from Jackson Laboratories. All experiments were reviewed and approved by the Institutional Animal Care and Use Committees of the University of Texas Medical Branch.

### Antibodies and Reagents

All flow cytometry antibodies and cytokines were obtained from BioLegend. CD4 magnetic beads were purchased from BD Biosciences. The CD25 MACS kit was manufactured by Miltenyi Biotec. TaqMan CCL25 and GAPDH primers were obtained from Applied Biosystems. The qScript cDNA SuperMix was obtained from Quanta. SsoAdvanced Universal SYBR Green Supermix was purchased from BioRad.

### Isolation of CD4^+^ and APCs

CD4^+^ T cells were isolated by using mouse anti-CD4 magnetic beads as previously described [6]. The mice were euthanized by CO2 inhalation followed by cervical dislocation. Mouse splenocytes were incubated with CD4 magnetic beads for 30 minutes at 4 C. Afterward, cells were placed on a magnetic column three times, for durations of 8, 4, and 4 minutes. After each incubation, the supernatant was aspirated, collected, and replaced by fresh buffer. The CD4^-^ fraction collected from the CD4+ isolation procedure was irradiated for use as antigen presenting cells (APCs).

### Isolation of Tregs

CD25^+^ T cells were tagged for isolation on a column using an anti-CD25 MACS kit. Cells were incubated with anti-CD25 PE-conjugated antibody for 15 minutes, washed via centrifugation, and then incubated with anti-PE magnetic beads for 5 minutes. Cells were isolated in a MACS sorter as described by the manufacturer’s protocol.

### Necropsy and Lamina Propria Lymphocyte (LPL) Isolation

The spleen, mesenteric lymph node (MLN), and small bowel (SB) and large bowel (LB) were extracted and fat and connective tissue carefully removed. The SB and LB were cleaned via scraping and cut into pieces, then were washed by shaking in PBS and straining through a sieve several times. Tissue was then incubated with magnetic stirrers in PBS with 1% FBS and 1:1000 EDTA at 37 C for 40 minutes to remove the epithelium. The remaining tissue was collected, washed in PBS several times, dried, and chopped finely. The tissue was then incubated with collagenase in RPMI with 2% FBS twice for 30 minutes at 37°C. Supernatant was collected, and fresh collagenase was added after the first incubation. After the second incubation, all of the supernatant was strained through a filter and washed via centrifugation. The pellet was then layered onto a 75%/40% percoll gradient and centrifuged, and the interface layer was collected for analysis.

### Preparation of Spleen, MLN, and Thymus Leukocytes

Single-cell suspensions were prepared from the spleen, MLN, and thymus by removing fat and connective tissue and smashing between glass slides. Leuckocyte suspensions were then washed via centrifugation and re-suspended. Additionally, splenic erythrocytes were then lysed with Tris-ammonia and the remaining leukocytes washed twice via centrifugation.

### Flow Cytometry

Intracellular and surface staining were performed as previously described [6]. For experiments in which intracellular cytokines were measured, cells were re-stimulated for 5 hours with PMA and ionomycin, with Golgi Stop being added for the last 3 hours of restimulation. Cells were washed via centrifugation and surface stain was added for 20 minutes at room temperature. Cells were then washed and permeablized for 30 minutes at 4°C by using a FoxP3 perm/fix kit manufactured by eBiosciences. After permeablization, cells were washed and incubated with intracellular antibodies for 30 minutes at 4°C before being washed a final time. Data were collected by using the LSRII/Fortessa and FACSDiva software. Compensation was performed by using FACSDiva at the time of collection. Further analysis was carried out by using FlowJo. All flow cytometry plots shown are first gated on lymphocytes using FSC and SSC, then gated on CD4-positive, live dye-negative cells.

### T Cell Culture and Polarization

Whole or naïve CBir1 Tg CD4^+^ T cells (1x10^5^) were isolated as described above and cultured in 24-well plates with irradiated APCs (1x10^5^) and 1 μg CBir1 peptide in RPMI with 10% fetal bovine serum for 5 days. Wild type (WT) APCs were used for all tissue culture experiments. In CCL25 experiments, cells were cultured with 2 ng/mL TGF-β to induce a Treg phenotype. Other Treg culture experiments utilized 10 ng/mL TGF-β. Retinoic acid (RA), where indicated, at a 1-μM concentration. A Th17 phenotype was induced by adding 10 ng/mL TGF-β, 30 ng/mL IL-6, 3.33 ng/mL anti-IFNγ, and 5 ng/mL anti-IL-4.

### Adoptive Transfer of CD4^+^ and CD25^+^ T Cells and Induction of Colitis

CBir1 CD4^+^ cells were transferred to TCRβxδ^-/-^ mice by tail vein injection. In Treg transfer experiments, Tregs were isolated by using a CD25 MACS kit as described above. All mice received equal numbers of Teff cells, and Tregs were transferred in a 1:1 ratio with Teffss. Mice were weighed weekly and examined for signs of disease. Mice were sacrificed and necropsy performed at 6 weeks post transfer, when symptoms of disease were evident.

### Histopathologic Assessment

During necropsy, sections of the LB and cecum were removed and Swiss rolls prepared. Tissue sections were fixed in 10% PFA overnight and embedded in paraffin. Sections were sliced to 5 μm, stained with H&E, and analyzed by a trained pathologist. The following histological features were analyzed: crypt epithelial hyperplasia, degeneration, and loss; mucosal ulceration; submucosal edema; LP and submucosal cellular infiltrate; transmural inflammation; crypt exudate; and goblet cell loss. Each component was scored separately for severity (0-absent, 1-mild, 2-moderate, 3-severe) and extent of tissue affected (0-absent, 1–25%, 2–50%, 3–75%, 4–100%), with the total score being the sums of the severity multiplied by the extent for each feature.

### Quantitative Real-Time PCR

RNA was quantified using two-step PCR. RNA was isolated from the small and large bowel using TRIzol reagent. cDNA was synthesized using qScript cDNA SuperMix and BioRad C1000 thermal cycler. Pre-designed TaqMan primers were used for cDNA synthesis. Quantitative real-time PCR was performed with the SsoAdvanced Universal SYBR Green Supermix using a BioRad CFX96 real-time PCR detection system. DNA amplification was detected with a SYBR green probe. Expression data was normalized to GAPDH mRNA levels and a designated control sample was arbitrarily given a value of 1.0.

### Statistical Analysis

Samples were analyzed in Prism (GraphPad) via Student’s T test. Paired tests were used where appropriate. The results were considered significant at a P value of less than 0.05.

## Results

### CCR9 Deficiency in Effector T Cells Does Not Alleviate Colitis Development

**Fig 1:**
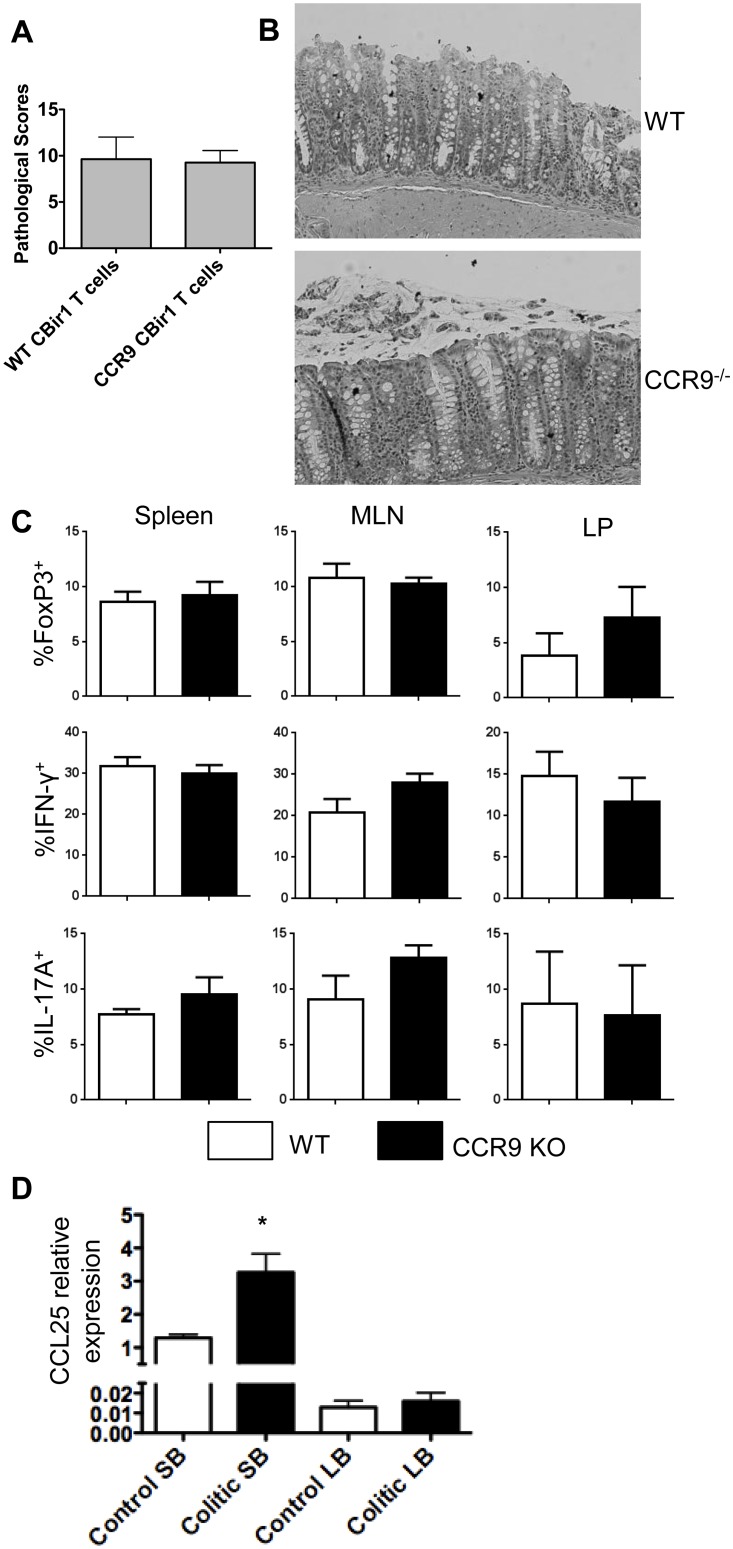
CCR9 deficiency in effector T cells does not affect colitis development.

Considering that blockade of CCR9 attenuates early intestinal inflammation [12,21], we first sought to examine the role of CCR9 in the pathogenesis of T cell-mediated colitis by using the CBir1-specific adoptive transfer model. We have previously shown that adoptive transfer of CBir Tg CD4^+^ T cells induces colitis in RAG^-/-^ or TCRβδ^-/-^ mice via *in vivo* generation of Th1 and Th17 Teff cells [6,30]. Colitis was induced by adoptive transfer of CD4^+^ T lymphocytes isolated from either WT CBir1Tg or CCR9^-/-^ CBir1 Tg mice into T cell-deficient TCRβδ^-/-^ mice. Mice were examined weekly and sacrificed once signs of disease became evident, which usually occurs at 6 weeks post T cell transfer. Histology samples were taken from the colon and cecum. Cytokine production by lymphocytes from the spleen, MLN, and lamina propria (LP) was measured via flow cytometry. Consistent with a previous report [31], there were no significant differences in pathology, IL-17 production, IFN-γ production, or FoxP3 expression in the spleen, MLN, or LP between WT and CCR9^-/-^ CD4^+^ T cell recipients (Fig 1A–1C). We then utilized quantitative real-time PCR to examine the expression of CCL25, the receptor for CCR9, in CBir1 CD4^+^ T cell recipient TCRβδ^-/-^ mice compared with control TCRβδ^-/-^ mice. We found robust expression of CCL25 in the SB, with only minimal CCL25 expression in the LB. In addition, CCL25 was upregulated in the small bowel of colitic mice compared to control mice, but not in the large bowel (Fig 1D). These data are in agreement with previous reports which found that CCL25 is primarily expressed in the small bowel and is upregulated under inflammatory conditions [18,32]. Hence, the similarity between the CCR9^-/-^ and WT CD4^+^ recipient groups cannot be explained by downregulation of CCL25 in our model. Collectively, these data indicate that CCR9 deficiency does not limit the capacity of Teff cells to induce disease in a T-cell mediated model of IBD.

Isolated CD4^+^ T cells (1x10^6^) from WT or CCR9^-/-^ CBir1 TCR transgenic mice were adoptively transferred to TCRβδ^-/-^ recipient mice. Colitis development was observed after six weeks, at which point the mice were sacrificed and necropsy performed. (A) Pathology was scored as described (B) and representative H&E-stained histopathology images from one experiment with 4 mice are shown. (C) Isolated lymphocytes from the spleen, MLN, and large intestine (LB) LP were stained for flow cytometry. Percentages of CD4^+^ T cells expressing IFN-γ, IL-17, and FoxP3 were determined by gating on live CD4^+^ populations and comparing relative expression. Averaged data from 2 experiments totaling 8 mice per group are shown. (D) CCL25 expression levels in the SB and LB of untreated TCRβδ^-/-^ mice were compared with those of CBir1 T cell recipient TCRβδ^-/-^ mice via quantitative real-time PCR. CCL25 expression levels are normalized to the reference gene GAPDH. The relative expression of CCL25 in the small intestines (SB) in control mice was arbitrarily set to 1.0. CCL25 expression was compared between the SB of colitic mice and the control SB. Data are representative of four mice per group. *P<0.01 compared with the control SB.

### CCR9 Deficiency in Tregs Does Not Affect Their Inhibitory Function during Colitis Development

**Fig 2:**
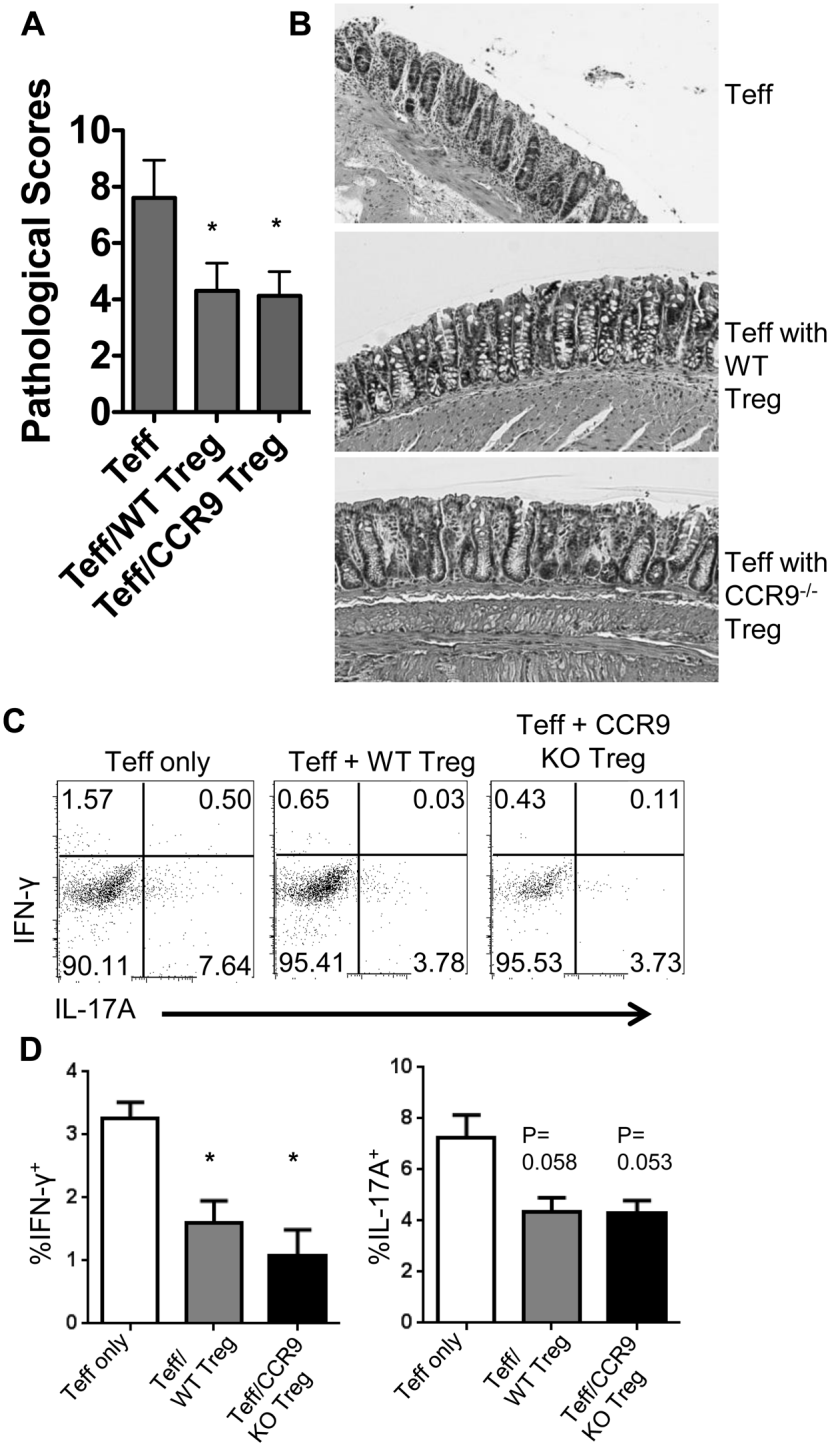
CCR9 deficiency in Tregs does not affect their ability to inhbit colitis.

We then sought to examine the effect of CCR9 deficiency in Tregs on their ability to suppress inflammation. Colitis was induced via adoptive transfer of CD4^+^ Teff cells isolated from CBir1 Tg mice into TCRβδ^-/-^ mice as described above. The recipient mice also received an equivalent number of CD25^+^ Tregs from WT or CCR9^-/-^ CBir1 Tg mice. Mice that received Teff cells but no Tregs served as positive controls. Mice were examined weekly and sacrificed once signs of disease became evident, generally at 6 weeks post transfer. We observed that mice which received WT or CCR9^-/-^ Tregs had lower pathology scores than did mice that received CBir1 Teff cells alone. However, mice which received CCR9^-/-^ Tregs had similar pathology scores to mice that received WT Tregs (Fig 2A and 2B). This finding indicates that CCR9^-/-^ Tregs had a similar capacity to inhibit CBir1 T cell-induced colitis as that of WT Tregs. T cell production of IFN-γ and IL-17 was decreased in mice which received WT Treg or CCR9^-/-^ Tregs compared to that in mice that received CBir1 Teffs alone (Fig 2C and 2D). These data indicate that loss of CCR9 in Treg cells does not impair their ability to control intestinal inflammation.

Purified WT CBir1 TCR transgenic CD4^+^ Teff cells (1 x 10^6^) were adoptively transferred to TCRβδ^-/-^ recipients to induce colitis. Two groups of mice also received 1 x 10^6^ of purified CD25^+^ CD4^+^ Treg cells from WT or CCR9^-/-^ CBir1 Tg mice in addition to the purified CD4^+^ T cells. (A)Pathology was scored as described. *P<0.01 compared with mice receiving Teff alone. (B) Representative H&E-stained histopathology images from one representative experiment of 3–4 mice are shown. (C-D)Lymphocytes were isolated from the LB LP and stained for flow cytometry. Percentages of CD4^+^ T cells in the LP expressing IFNγ and IL-17 were determined by gating on live CD4^+^ populations and comparing relative expression. Representative FACS plotsand bar charts from one representative experiment of 3–4 mice per group are shown. Data are reflective of 2 independent experiments. *P<0.05 compared with the mice receiving Teff alone.

### Treg Cells Express Higher Levels of CCR9 Compared to Effector T Cell

**Fig 3:**
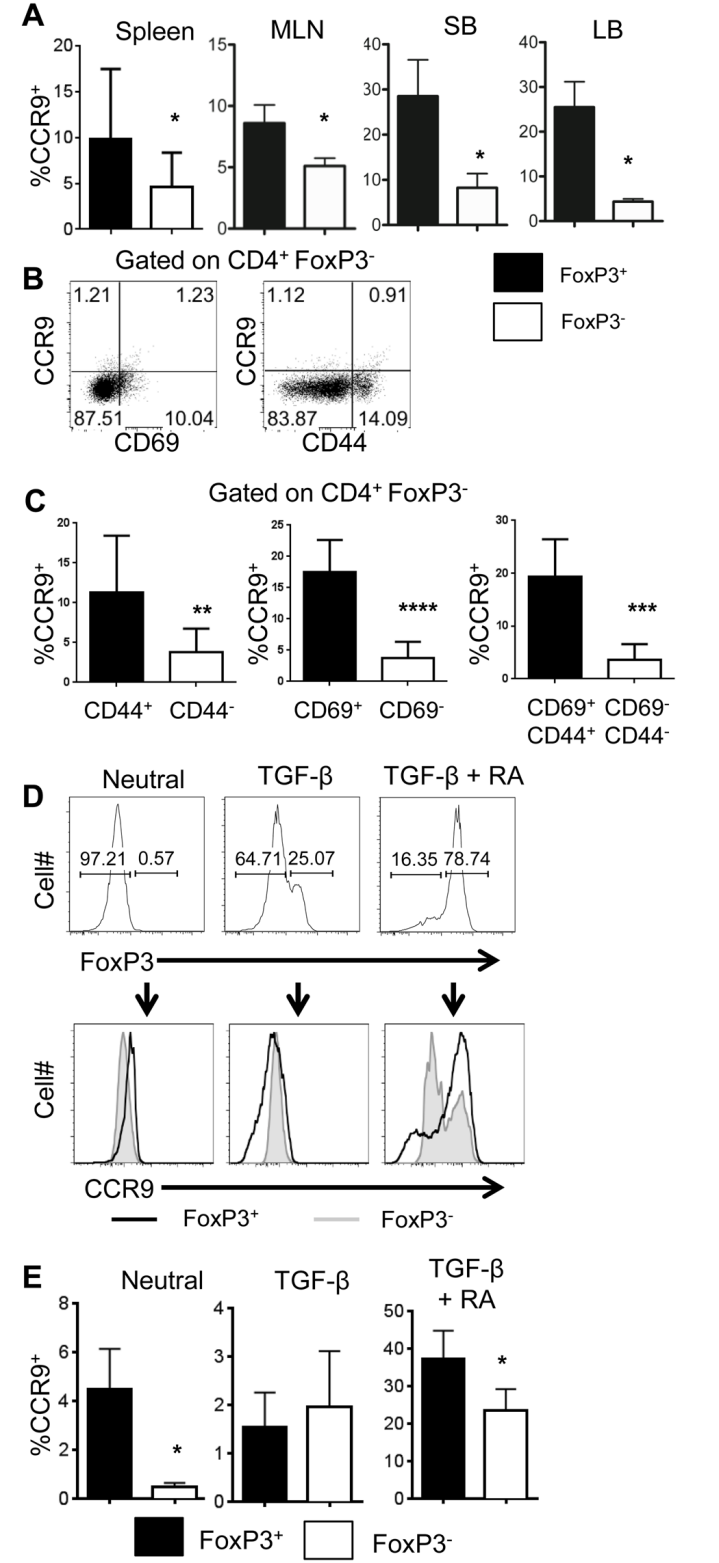
CCR9 is preferentially expressed by Tregs.

We then examined CCR9 expression on Teffs and Tregs. WT CBir1 tg mice were sacrificed, and lymphocytes from the spleen, MLN, SB, and LB were harvested and stained for flow cytometry. We found that expression of CCR9 on CD4^+^ T cells was enriched on the FoxP3^+^ fraction when compared with the FoxP3^-^ fraction in the spleen, MLN, SB and LB (Fig 3A). We then examined CCR9 expression on naïve and activated CD4^+^ T cells, as measured by expression of T cell memory and activation markers CD44 and CD69, in relation to CCR9 expression. We first gated on the FoxP3^-^ population, then examined co-expression of CCR9 with CD44 and CD69 in FoxP3^-^ CD4^+^ T cells. To obtain statistical data, we gated the CD4^+^ FoxP3^-^ population into three sets representing the activated and naive phenotypes, respectively: CD44^+^ versus CD44^-^, CD69^+^ versus CD69^-^, and CD44^+^CD69^+^ versus CD44^-^CD69^-^. The percent expression of CCR9 in each of the activated and non-activated FoxP3^-^ populations was then determined. We observed that CCR9 was expressed primarily on activated memory and effector T cells expressing CD44 and/or CD69. We also observed CCR9 expression on CD44^-^ and CD69^-^ naive T cell populations, albeit at low levels (Fig 3B and 3C). We then set out to confirm these findings *in vitro* by culturing isolated WT CD4^+^ T cells with or without TGF-β, or with TGF-β and RA. Consistent with previous reports [33], RA induced T cell expression of CCR9. Interestingly, FoxP3^+^ T cells expressed higher levels of CCR9 than FoxP3^-^ cells from the same culture (Fig 3D and 3E). This finding suggests that CCR9 is preferentially upregulated on Treg cells via an RA-dependent, TGF-β-independent mechanism.

Spleen, MLN, SB LP and LB LP CD4+ T cells were harvested from healthy WT and CCR9^-/-^ mice and prepared for flow cytometry as described. (A) Live CD4^+^ T cells were gated into FoxP3- positive and -negative fractions and analyzed for expression of CCR9. *P<0.05 compared with Foxp3^+^ cells. (B) Expression of CCR9 in relation to activation status, measured as an expression of CD44 and CD69, was examined on CD4^+^ FoxP3^-^ effector T cells. CCR9 expression in relation to CD44 and CD69 is shown by using representative FACS plots. (C) Statiscal analysis of CCR9 expression by activation status was was performed by comparing the percent CCR9^+^ cells in the following CD4^+^FoxP3^-^ populations: CD44^+^ versus CD44^-^, CD69^+^ versus CD69^-^, and CD44^+^CD69^+^ versus CD44^-^CD69^-^ and bar charts illustrative of 7 mice. **P<0.01; ***P<0.001; ****P<0.0001. (D-E) Isolated CD4^+^ T cells from CBir1 Tg mice were cultured under neutral conditions, with TGF-β, or with TGF-β and RA and examined via flow cytometry. CD4^+^ T cells were gated into FoxP3^+^ and FoxP3^-^ compartments, and expression of CCR9 in these populations was compared. Representative FACS profiles and bar charts indicating mean percent expression from 3 experiments totaling 9 samples are shown. *P<0.05 compared with Foxp3^+^ cells.

### CCR9^-/-^ Mice Demonstrate Enrichment of Foxp3^+^ Tregs

**Fig 4:**
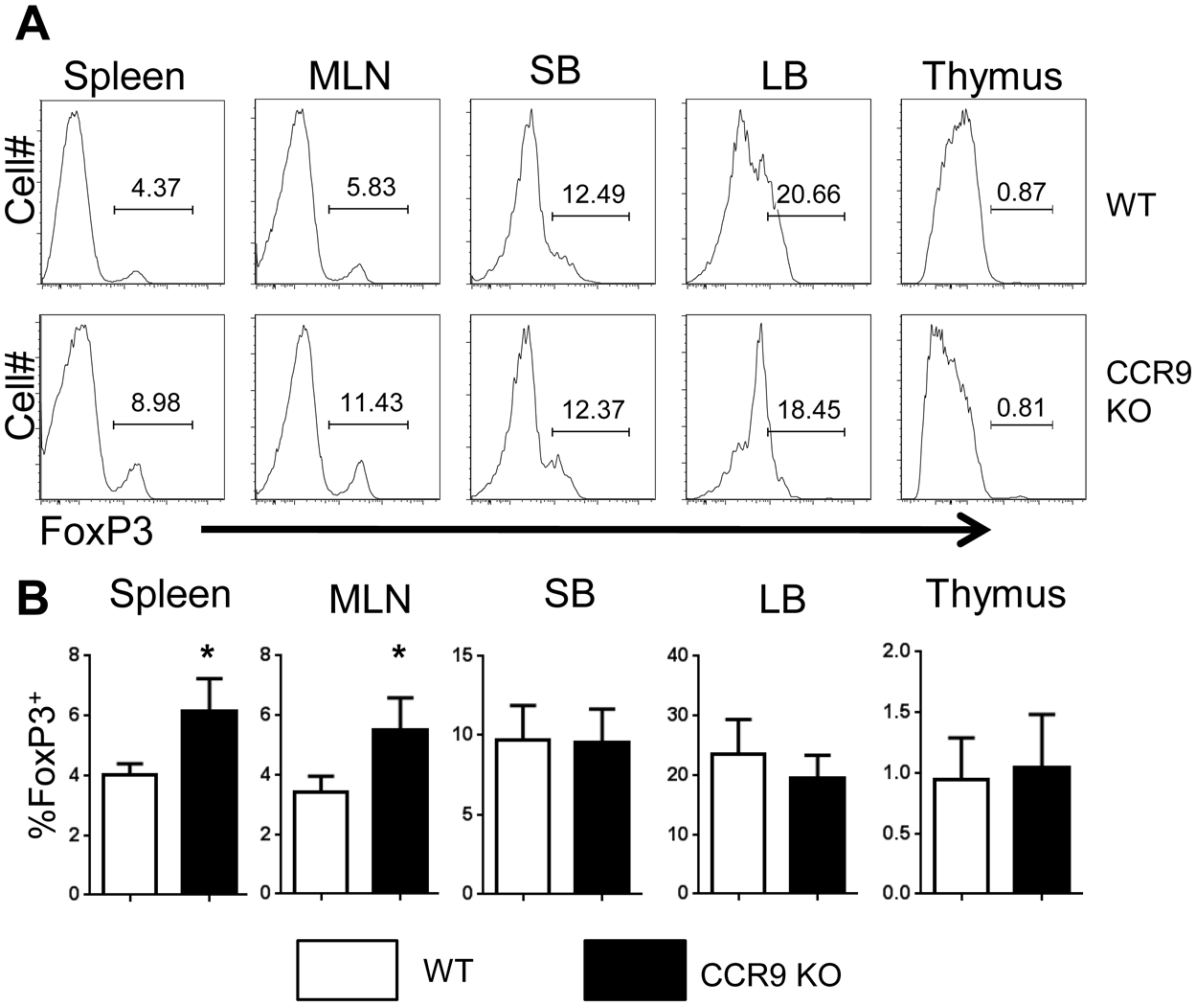
CCR9 deficiency results in enhanced Treg populations.

**Fig 5:**
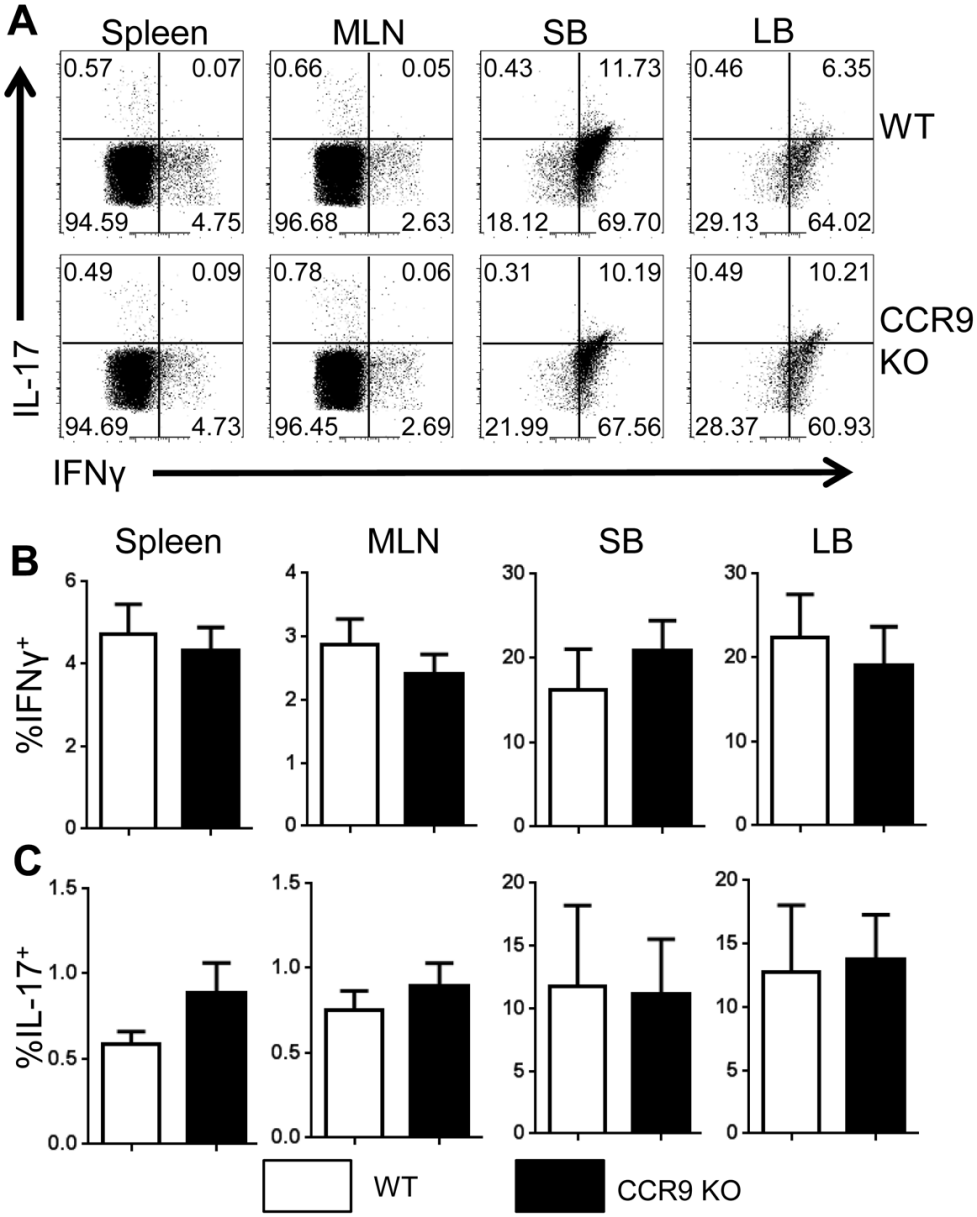
Deficiency in CCR9 does not affect Th1 or Th17 cell populations *in vivo*.

To determine whether CCR9 deficiency affects Treg cell development, WT mice and CCR9^-/-^ mice were sacrificed, and lymphocytes from the spleen, MLN, SB, and LB were harvested and stained for flow cytometry. We found an increase in Foxp3^+^ Tregs in the spleen and MLN of CCR9^-/-^ mice compared to that in WT mice. However, there was no significant difference in the number of intestinal Tregs in CCR9^-/-^ mice compared to that in WT mice (Fig 4A and 4B). This is possibly due to impaired Treg migration to the intestine in CCR9^-/-^ mice, as there were more Tregs in the periphery. To determine if CCR9 deficiency specifically regulates induced Treg (iTreg) or natural Treg (nTreg), we also analyzed Treg cell expression in the thymus. Interestingly, we observed no difference in FoxP3 expression by CD4^+^ CD8^-^ thymocytes in WT vs CCR9^-/-^ mice (Fig 4A and 4B), indicating that nTreg differentiation is not impaired. These data suggest that CCR9 differentially regulates the development of iTregs and nTregs by inhibiting the differentiation of iTregs in the periphery. There was no change in CD4^+^ T cell production of IFN-γ and IL-17 in CCR9^-/-^ mice, indicating that CCR9 signaling has no impact on Th1 or Th17 Teff populations (Fig 5).

Spleen, MLN, SB LP and LB LP CD4^+^ T cells were harvested from WT and CCR9^-/-^ mice and stained for flow cytometry as described. (A-B) Cells were gated on live CD4^+^ lymphocytes and the percentage of FoxP3^+^ Tregs out of the total CD4^+^ cells was analyzed. Representative FACS profiles (A) and average percentage of FoxP3 expression (B) data from 10 mice per group are shown. *P<0.05 compared with wild-type mice.

Spleen, MLN, SB LP and LB LP CD4^+^ T cells were harvested from naïve WT and CCR9^-/-^ mice and stained for flow cytometry as described. (A) Cells were gated on live CD4^+^ lymphocytes, and the percentage of CD4^+^ cells expressing IFNγ and IL-17 was analyzed. Representative FACS plots are shown. (B-C) Mean percent expression data for IFNγ and IL-17 were also analyzed. Data from 3 experiments totaling 9 mice are shown.

### CCR9-CCL25 Interaction Inhibits Treg Differentiation

**Fig 6:**
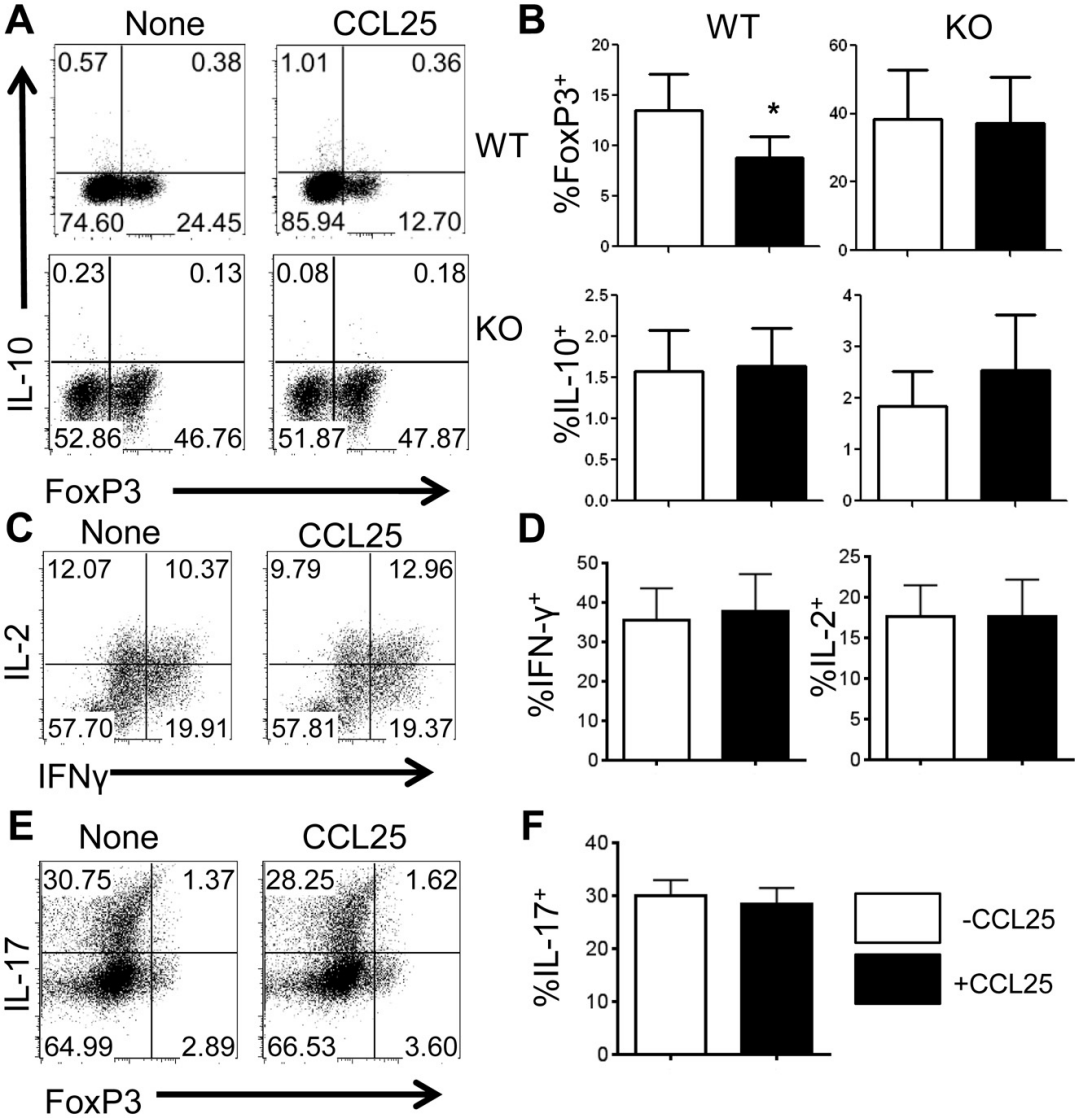
Ligation of CCR9 by CCL25 inhibits Treg development.

To further determine the effect of CCR9 on Treg differentiation, we investigated whether binding of CCR9 to its ligand CCL25 inhibits Treg cells. We cultured total spleen CBir1 Tg CD4^+^ T cells under Th1, Th17 and Treg conditions in the presence or absence of CCL25. About 3% of the CD4^+^ T cells were Foxp3^+^ prior to culture (data not shown). Interestingly, addition of CCL25 inhibited FoxP3^+^ Tregs when cultured with TGFβ, which was not observed in CCR9^-/-^ CD4^+^ T cells (Fig 6A and 6B). These data indicate that inhibition of Treg is directly dependent on CCR9-CCL25 interaction. CCL25 treatment did not affect IL-10 production. We also examined the effect of CCR9 signaling via CCL25 ligation on other CD4^+^ T cell subsets. Under neutral culture conditions, treatment with CCL25 did not affect the production of IL-2 or Th1 generation as measured via IFNγ (Fig 6C and 6D). Likewise, CCL25 treatment had no effect on IL-17 production under Th17-polarizing conditions (Fig 6E and 6F). These data indicate that CCR9 signaling does not affect the generation of Th1 and Th17 cells.

Purified WT and CCR9^-/-^ CD4^+^ T cells were cultured under various T helper-polarizing condtions with or without CCL25 (20 ng/mL). Cells were stained for flow cytometry as described and gated on live CD4^+^ lymphocytes, which were then analyzed for the percentage of expression of FoxP3, IFNγ, IL-17, and IL-10. (A-B) Isolated CD4^+^ T cells cultured under Treg- polarizing conditions were analyzed for expression of FoxP3 and IL-10.Representative FACS plots and mean percentage of expression calculated from 4 independent experiments, are shown. *P<0.05 compared with cells cultured in the absence of CCL25. (C-D) Cells cultured under neutral conditions were analyzed for IFN γ and IL-2, with representative FACS plots and average percentage of expression from 6 independent experiments shown. (E-F) Cells cultured under Th17-polarizing conditions were analyzed for IL-17, with representative FACS plots and mean percentage of expression from 4 independent experiments shown.

## Discussion

Our current study unexpectedly demonstrated that in addition to acting as a homing molecule to the small intestines, CCR9 inhibits Treg cell development. The inhibition of Treg expansion by CCR9 ligation to CCL25 may indicate that CCR9 contributes to the uncontrolled inflammation seen in IBD by inhibiting Treg development. CCR9, as a lymphocyte homing factor to small intestines, has been extensively investigated as a potential therapeutic target in IBD, although controversial results have been generated from various studies. CCR9 expression is increased in patients with IBD and has been shown to regulate colitis development in various animal models [12,15,19,22,31,34]. However, the mechanisms involved are still not completely understood. In a DSS-induced colitis model, CCR9^-/-^ mice developed more severe colitis compared to that in WT mice upon insults with DSS. CCL25^-/-^ Rag^-/-^ mice developed more severe disease compared to that in WT Rag^-/-^ mice on transfer of CD45RB^hi^ T cells [31], possibly meaning that innate CCR9 expression also regulates colitis development. It has been shown recently that CCR9 mediates both Teff cell and Treg cell trafficking to the SB, but not to the LB [12,15,35].

CCR9 expression on naïve T effector cells or on Treg cells is not required for induction or regulation of colitis [31]. Consistently, when we transferred microbiota antigen-specific T cells from CBir1 Tg mice into Rag^-/-^ mice, CCR9-deficient T cells were able to induce equally severe of colitis when compared to that induced by WT T cells. Furthermore, CCR9-deficient Treg cells demonstrated suppressive function similar to that of WT Treg cells in our model, confirming the notion that CCR9 expression is not required for Teff or Treg cell migration and function in the LB.

Although most investigations have been focused on the role of chemokines in promoting lymphocyte migration processes in various tissues during homeostasis or inflammation, recent studies also demonstrated distinct, non-chemoattractant functions of chemokines in directly regulating T cell differentiation and function. In addition to being a highly efficient and potent chemoattractant for T cells into specific tissues [36], CXCL12 can promote T cell expression of IL-10 to differentiate into IL-10-producing Tr1 cells, thus functioning as an anti-inflammatory chemokine [25]. CXCL11 does not only promote naïve T cell differentiation into IL-10-producing Tr1 cells, but is also able to convert CXCR3^+^ effector T cells into Tr1 cells during the development of EAE. In contrast, CXCL10 was reported to promote Th1 cell development [26].

In our current study, we found that CCR9 expression was higher in Treg cells compared to that in T effector cells *in vivo*. Although these data are consistent with previous studies that indicate RA induces T cell expression of CCR9, our data also indicated that the CCR9 expression was higher in Foxp3^+^ T cells when compared to findings in Foxp3^-^ T cells after treatment with RA *in vitro*. CCR9 deficiency appeared to promote Foxp3^+^ Treg cell development *in vivo*, with CCR9^-/-^ mice demonstrating enriched Foxp3^+^ Treg populations compared to WT mice. Furthermore, ligation of CCR9 with CCL25 *in vitro* resulted in fewer Foxp3^+^ Tregs in CD4^+^ T cell cultures treated with TGF-β, indicating that CCR9 signaling inhibits Foxp3^+^ Treg cell development. We still do not understand the mechanisms mediating CCR9-CCL25 inhibition of Treg cells. As naïve CD4 T cells express very low levels CCR9, we speculate that CCR9 signaling may inhibit Treg cell survival and/or proliferation. Therefore, our study strongly supports the notion that in addition to its well-established function of mediating T cell migration to certain specific tissues, signaling via chemokine receptors can also directly regulate T cell function.

Current clinical trials using a CCR9 antagonist, CCX282-B, which inhibits CCR9- and CCL25-dependent chemotaxis, reported only minimal efficacy[12,18,19]. Our data suggest that development of reagents inhibiting CCR9 signaling to promote Treg development, in addition to blocking effector T cell migration to inflamed sites of the intestines, warrants consideration.
